# A comparison of the association between large haplotype blocks under selection and the presence/absence of inversions

**DOI:** 10.1002/ece3.5094

**Published:** 2019-03-27

**Authors:** Sangeet Lamichhaney, Leif Andersson

**Affiliations:** ^1^ Department of Organismic and Evolutionary Biology and Museum of Comparative Zoology Harvard University Cambridge Massachusetts; ^2^ Department of Medical Biochemistry and Microbiology Uppsala University Uppsala Sweden; ^3^ Department of Veterinary Integrative Biosciences Texas A&M University College Station Texas; ^4^ Department of Animal Breeding and Genetics Swedish University of Agricultural Sciences Uppsala Sweden

**Keywords:** adaptation, haplotypes, inversion

## Abstract

Inversions may contribute to ecological adaptation and phenotypic diversity, and with the advent of “second” and “third” generation sequencing technologies, the ability to detect inversion polymorphisms has been enhanced dramatically. A key molecular consequence of an inversion is the suppression of recombination allowing independent accumulation of genetic changes between alleles over time. This may lead to the development of divergent haplotype blocks maintained by balancing selection. Thus, divergent haplotype blocks are often considered as indicating the presence of an inversion. In this paper, we first review the features of a 7.7 Mb inversion causing the Rose‐comb phenotype in chicken, as a model for how inversions evolve and directly affect phenotypes. Second, we compare the genetic basis for alternative mating strategies in ruff and timing of reproduction in Atlantic herring, both associated with divergent haplotype blocks. Alternative male mating strategies in ruff are associated with a 4.5 Mb inversion that occurred about 4 million years ago. In fact, the ruff inversion shares some striking features with the Rose‐comb inversion such as disruption of a gene at one of the inversion breakpoints and generation of a new allele by recombination between the inverted and noninverted alleles. In contrast, inversions do not appear to be a major reason for the fairly large haplotype blocks (range 10–200 kb) associated with ecological adaptation in the herring. Thus, it is important to note that divergent haplotypes may also be maintained by natural selection in the absence of structural variation.

## 1 INTRODUCTION

An inversion represents the breakage of a chromosome at two points and reinsertion of the segment bound by the breakpoints in the reversed orientation (Sturtevant, 1921). An inversion can change the order of genes along the chromosome and/or reorganize regulatory domains leading to altered gene expression (Puig, Casillas, Villatoro, & Cáceres, 2015). As a result, inversions can directly become associated with a phenotypic change (Ayala et al., 2011). Pioneering population‐level studies on the significance of inversions were documented based on polytene chromosomes in *Drosophila*, starting from the work of Dobzhansky & Sturtevant (1938). The *Drosophila* system has provided rich evidence that inversion polymorphisms may be under strong selection and contribute to adaptation in natural populations (Krimbas & Powell, 1992).

Although inversion was one of the first population genetic markers to be used (Hoffmann & Rieseberg, 2008), they gradually fell from the focus of researchers in the last few decades, mostly as a result of the challenges in detecting them. Inversions were first identified using polytene chromosomes preparations (Painter, 1933) and then later using cytogenetic techniques such as G‐banded karyotypes (Feuk, 2010) or fluorescence in situ hybridization (Antonacci et al., 2009). But these methods were limited to identification of inversions that were several megabases in size, and methods to detect relatively smaller inversions were lacking.

In recent years, next‐generation sequencing methods have been extensively used for the global detection of sequence polymorphisms (including inversions) directly from the genomes of many nonmodel species. Next‐generation paired‐end reads provide a sequence‐based method for identification and local de novo assembly of inversion breakpoints (Corbett‐Detig, Cardeno, & Langley, 2012). In addition, methods that utilized paired‐end sequencing reads from multiple individuals (and populations) to search for inversions based on abnormal orientation mappings to the reference genome were developed (Escaramis, Docampo, & Rabionet, 2015). This technological and methodological advancements have led to the identification of inversions associated with phenotypic variation in multiple species (Wellenreuther & Bernatchez, 2018). However, a major challenge is still the molecular characterization of the exact inversion breakpoints, in particular if they are located in repetitive regions.

A key feature of inversions is that they suppress recombination within the inverted region leading to independent accumulation of additional genetic changes and thereby sequence divergence between “wild‐type” and inverted chromosomes (Kirkpatrick, 2010). This allows for the development of divergent haplotype blocks where certain combinations of allelic variants at multiple sites have a higher fitness than random combinations of variants. Detection of divergent haplotype blocks are often interpreted as suggestive evidence for the presence of inversions (Jaarola, Martin, & Ashley, 1998).

However, divergent haplotype blocks may not necessarily indicate the presence of an inversion. The synergistic effect of variants at multiple sites is well illustrated by the stepwise evolution of alleles in domestic animals (Andersson, 2013). Despite their relatively short evolutionary history, there are a number of examples where phenotypic traits in domestic animals are caused by such combined effects of multiple mutations affecting the same gene—for example, dominant white coat color in pigs (Marklund et al., 1998), black spotting in pigs (Fang, Larson, Ribeiro, Li, & Andersson, 2009), and sex‐linked barring in chicken (Schwochow Thalmann et al., 2017). Such linked mutations may affect the coding sequence or gene regulation and can therefore lead to the generation of divergent haplotype blocks, spanning at least hundreds of kilobases. This is because regulatory domains affecting the same gene may be located hundreds of kilobases away from the coding sequence. As an example, a 20‐kb duplication is causing the Duplex‐comb phenotype in chicken by driving ectopic expression of the *EOMES* gene, despite the fact that the duplication is located within an intron of another gene located 200 kb upstream of *EOMES *(Dorshorst et al., 2015). Hence, an important question is whether inversions is or is not a common explanation for divergent haplotype blocks under selection in natural populations.

Here, we have compared the data from our previously published studies that have identified genomic regions associated with adaptive traits in two different species systems as follows: (a) alternative mating strategies in ruff (Lamichhaney, Fan et al., 2016) (b) reproductive strategies in Atlantic herring (Lamichhaney et al., 2017; Martinez Barrio et al., 2016). We had used a similar approach to explore the underlying molecular basis of particular adaptive trait in each of these systems, and the commonality was the presence of divergent haplotype blocks associated with phenotypic variation. However, we start by reviewing the *Rose‐comb* locus in chicken as a model for direct phenotypic changes caused by inversions and for the evolution of inversions. The reason for this is that inversions maintained in natural populations may reflect a long evolutionary history, and the strong linkage disequilibrium within the inversion makes it exceedingly difficult to dissect to which extent phenotypic changes associated with an inversion polymorphism is caused by the inversion itself or genetic changes that have accumulated subsequent to the inversion event. The Rose‐comb inversion is most likely very recent, subsequent to domestication of chicken, and therefore provides an opportunity to explore the direct consequences of an inversion.

## 2 THE *ROSE‐COMB* LOCUS IN CHICKEN—HOW INVERSIONS EVOLVE AND AFFECT PHENOTYPES

**Figure 1:**
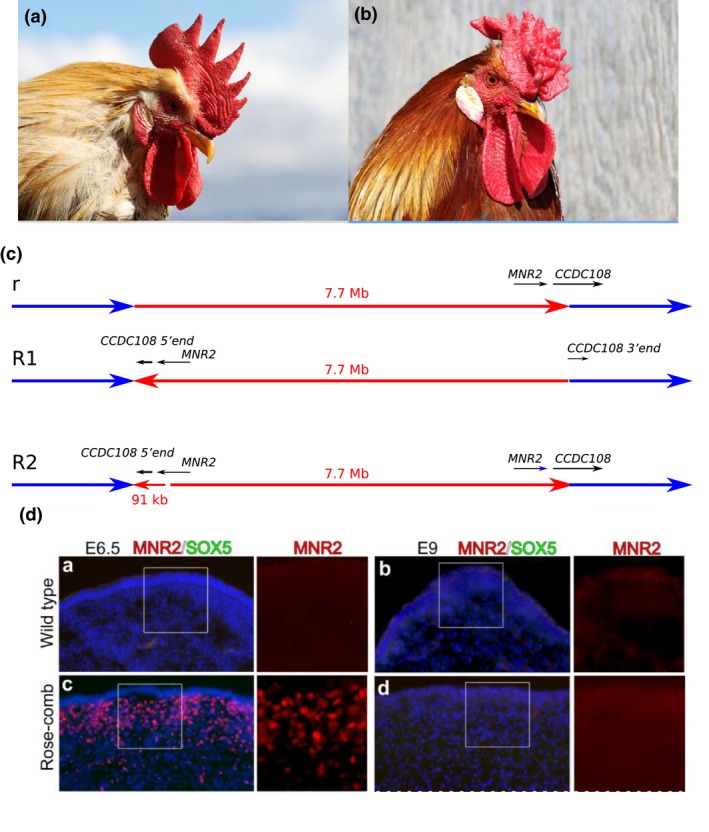
The *Rose‐comb* locus in chicken. (a) Single‐combed wild‐type male *r/r*. (b) Rose‐combed male *R/*‐. (c) Schematic illustration of the three alleles, *wild‐type—r* (upper panel), *R1* (middle panel), and *R2* (lower panel). The locations of the *CCDC108* and *MNR2* genes in relation to the inversion break points are indicated. The small arrow representing the 91‐kb region containing the translocated 5′ end of CCDC108 and MNR2 is drawn out of scale. (d) Immunohistochemical labeling of MNR2 and SOX5 in wild‐type single comb (a, b) and Rose‐comb (c, d) sections from embryonic day (e) 6.5 (a, c) and 9 (b, d). Figures (a, b), and (d) from (Imsland et al., 2012)

*Rose comb* is one out of the three major loci affecting comb morphology in chickens. The *Rose‐comb* (*R*) allele is fully dominant over *wild‐type* (*r*) as regards its effect on comb morphology (Figure 1a,b). Rose comb was one of the traits included in Bateson's seminal paper from 1902 demonstrating Mendelian inheritance in animals for the first time (Bateson, 1902). *Rose comb* has two distinct phenotypic effects, a dominant effect on comb morphology and males that are homozygous *R/R* show low fertility due to reduced sperm motility (Crawford, 1965). This association between comb morphology and male fertility was a mystery until the causal mutation was revealed (Imsland et al., 2012) as reviewed here.

Classical pedigree‐based linkage mapping assigned the *Rose‐comb* locus to chicken chromosome 7, and it was noted that recombination was severely suppressed in *R/r* heterozygotes. The recombination frequency over an ~8 Mb region was 0% and genetic markers in this region showed 100% linkage disequilibrium on *Rose‐comb* chromosomes across breeds (Imsland et al., 2012). Whole‐genome sequencing (WGS) using mate‐pair libraries (average insert sizes in the range 2.6–3.9 kb) of three pools of chickens representing two different breeds with the Rose‐comb phenotype and one with the wild‐type phenotype revealed the presence of a 7.7 Mb inversion on the *Rose‐comb* chromosome (Figure 1c). Interestingly, the WGS analysis revealed two different *Rose‐comb* alleles denoted *R1* and *R2* (Figure 1c). *R1* is composed of an intact inversion while *R2* must be the product of a nonhomologous recombination event between (a) a sequence on an *r* (wild‐type) chromosome located just outside where the inversion breakpoint is and (b) a sequence 91 kb inside the inversion on the *R1* allele. *R2* is identical to the wild‐type *r* allele except for this 91‐kb fragment of the inverted chromosome at the proximal breakpoint. Thus, this 91‐kb fragment is duplicated in the *R2* allele, it occurs in its wild‐type configuration at the distal breakpoint and in the inverted orientation at the proximal breakpoint (Figure 1c). This illustrates the merit of using domestic animals in studies of an evolutionary process. Due to the short evolutionary history, it is often possible to identify intermediate steps in the evolution of an allele like *R2* that is the result of multiple consecutive mutations.

Because the comb phenotypes associated with the *R1* and *R2* alleles are indistinguishable, the causal change should be found in the translocated 91‐kb fragment shared by these two alleles. Functional studies revealed that the causal change is ectopic expression of the transcription factor MNR2 homeodomain protein (Imsland et al., 2012). Immunohistochemical labeling of MNR2 revealed transient ectopic expression during development in a layer of mesenchymal cells located in the area where the comb is developing (Figure 1d). This ectopic expression was present at day 6.5 but not at day 9 of embryonic development. Interestingly, also the other two comb phenotypes in chicken, Pea‐comb and Duplex‐comb, are caused by structural changes (a copy number expansion and a duplication, respectively) in or in the vicinity of two other genes (*SOX5 *and *EOMES*, respectively) leading to a very similar ectopic expression of these transcription factor genes (Dorshorst et al., 2015; Wright et al., 2009). In fact, there is a clear overrepresentation of structural changes (duplications, deletions, and inversions) underlying visible phenotypes in domestic animals, and this list includes in addition to the three comb phenotypes for instance fibromelanosis in chicken, graying with age in horses, dominant white color in pigs, and a dorsal hair ridge in dogs (Andersson, 2013).

The fact that *R1/R1* but not *R2/R2* homozygous males showed reduced fertility implied that this phenotype must be caused by a genetic alteration at the distal breakpoint that is present in *R1* but not in *R2 *(Imsland et al., 2012). The obvious candidate change is the disruption of *CCDC108* (*coiled‐coil domain containing 108*), a gene with deep roots in the tree of life. This notion is strongly supported by the facts that (a) this gene is expressed in testis, (b) the CCDC108 protein contains an MSP (major sperm protein) domain shared among sperm proteins and (iii) the orthologous protein in *Chlamydomonas* algae is named Flagellar Associated Protein 65. Thus, it appears highly likely that the disruption of *CCDC108* is causing reduced sperm motility in *R1/R1* homozygous males. The results predicted that *CCDC108*, now renamed as *Cilia and flagella associated protein 65* (*CFAP65*), is a candidate gene for sperm motility disorders in humans (Imsland et al., 2012). A prediction that in fact was recently confirmed in human studies (Tang et al., 2017).

The *Rose‐comb* inversion illustrates (a) how new alleles may evolve by recombination between a noninverted and an inverted allele, (b) how an inversion can alter the expression pattern of a gene although its coding sequence is not touched by the inversion and (c) how an inversion breakpoint may disrupt a coding sequence. Furthermore, it shows that a phenotype caused by ectopic gene expression may show dominant inheritance because it acts as a gain‐of‐function (expression in a cell population where the gene normally is not expressed), whereas inactivation of a gene often shows a recessive inheritance (Lodish et al., 2000) because expression of a single functional copy are sufficient for most genes, as well documented by the fact that inactivation of most human genes do not show a disorder or visible phenotype in heterozygotes.

## 3 ALTERNATIVE REPRODUCTIVE STRATEGIES IN MALE RUFF ARE ASSOCIATED WITH A CHROMOSOMAL INVERSION

**Figure 2:**
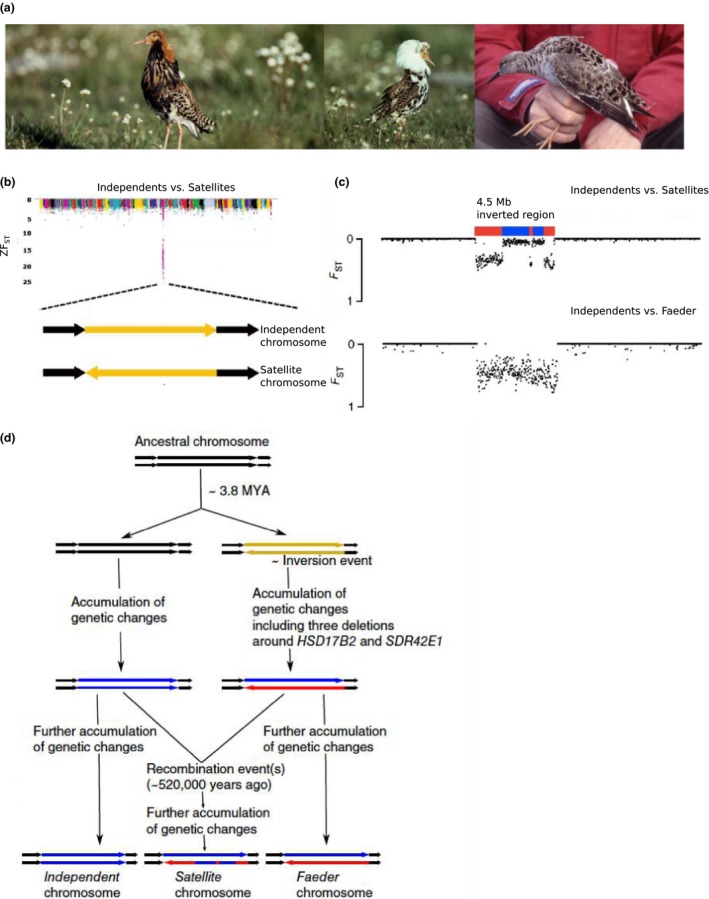
A 4.5 Mb inversion locus underlying alternative mating strategies in ruff. (a) Three different male morphs, Independent (left), Satellite (center), and Faeder (right). (b) Normalized *F*_ST_ scores in 15‐kb windows indicates a 4.5 Mb region showing strong differentiation between Independents and Satellites (upper panel). The region overlaps an inversion present in Satellites (lower panel) and Faeders. (c) Genetic differentiation (*F*_ST_) between Independents and Satellites, and between Independents and Faeder, around the inversion locus. (d) Evolutionary history of the three alleles underlying different male morphs. Figures modified from (Lamichhaney, Fan et al., 2016)

The ruff *(Calidris pugnax) *is a medium‐sized wading bird that breeds in marshes and wet meadows in the palearctic zone. Three different male morphs (Independents, Satellites and Faeders) show remarkable differences in breeding plumage, size, and male mating strategies (Lank & Dale, 2001) (Figure 2a). Each of these three male morphs uses unique courtship behavior to reproduce and survive. Independents (80%–95% of males) show spectacular diversity in color of the ruff and head tufts, and vigorously defend territories. Satellites (5%–20% of males) usually have white ruff/head tufts, do not defend territories and display a submissive behavior against the Independent males. Faeder is a rare (<1% of males) third morph, mimicking females by its smaller size, and female‐like plumage. Independents attract females by their elaborate performance display, high degree of aggression and strongly colored plumage. Satellites allow Independents to dominate them, in exchange of getting closer to females visiting the territories occupied by Independents on the lek. Faeder being a female mimic, gets access to mating territories in disguise and attempt to mate females whenever there is an emerging opportunity (See video clip at https://www.scilifelab.se/news/a-supergene-underlies-genetic-differences-in-testosteron-levels-and-sexual-behaviour-in-male-ruff/). In our previous study, we generated a genome assembly from an Independent male and performed whole‐genome sequencing of 15 Independents, nine Satellites, and a single Faeder male (Lamichhaney, Fan et al., 2016). The sequence reads were mapped to the Independent reference genome, and SNP data were generated. Furthermore, estimation of average *Z*‐normalized fixation index (ZF_ST_) in each 15‐kb window across the genome between Independents and Satellites identified a strongly differentiated 4.5‐Mb region (ZF_ST_ > 10; Figure 2b) between these two populations. As a follow up to the *F*_ST_ analysis, we screened the entire genomes of Independent, Satellites, and a single Faeder to identify structural variants using Breakdancer (Fan, Abbott, Larson, & Chen, 2014) and identified a heterozygous inversion in Satellites and the Faeder that overlapped the strongly differentiated 4.5‐Mb region (ZF_ST_ > 10; Figure 2b; Lamichhaney, Fan et al., 2016). The 5′ end of the inversion disrupts *CENPN*, a gene that encodes centromere protein N and its inactivation has severe deleterious effect in other species (Amsterdam et al., 2004; Foltz et al., 2006). Therefore, we hypothesized that this inversion is recessive lethal and hence maintained by balancing selection. A recent study on pedigrees from a captive ruff population has confirmed that this inversion is in fact recessive lethal (Kupper et al., 2015).

In our previous study, we also computed the time since divergence among these three morphs which indicated that inversion must have occurred ~3.8 million years ago (Lamichhaney, Fan et al., 2016). Furthermore, the average nucleotide sequence divergence (~1.4%) between Independent and Faeder chromosomes was consistent with an inversion that completely suppress recombination, as the sequence divergence was equally strong across the inverted region (Figure 2c). In contrast, the sequence divergence between the Independent and Satellite chromosomes showed a peculiar pattern with variable levels of sequence divergences (Figure 2c). The most likely explanation for this observation is that one or two double recombination events have occurred between these chromosomes. We estimated that these events happened ~500,000 years ago based on the low sequence divergence regions. Figure 2d summarizes our interpretation of the evolutionary history of the inversion locus in ruff. We hypothesized that the genetic differences between the three male morphs are to a large extent the result of the accumulation of many sequence changes that increase the fitness of the different alleles. One example of sequence differences that are likely to affect function is the presence of three deletions affecting evolutionary conserved noncoding sequences in the vicinity of *HSD17B2*, a gene encoding an enzyme with a critical role for the metabolism of testosterone since it converts active testosterone to an inactive keto‐form. An upregulated expression of this gene may explain the strikingly low levels of circulating testosterone in Satellite and Faeder males (Kupper et al., 2015). This finding of how an ancestral chromosomal inversion can trigger an evolution of a large divergent haplotype (“supergene”) in ruff is consistent with recent findings in *Heliconius* (Jay et al., 2018) and *Drosophila* (Fuller, Leonard, Young, Schaeffer, & Phadnis, 2018). It is also worth noting the striking similarities between the ruff and *Rose‐comb* inversions as regards gene inactivation at an inversion breakpoint and how new alleles with distinct phenotypes have emerged due to recombination between inverted and noninverted alleles.

## 4 INVERSIONS ARE NOT A MAJOR REASON FOR LARGE HAPLOTYPE BLOCKS ASSOCIATED WITH SPAWNING DIVERSITY IN ATLANTIC HERRING

**Figure 3:**
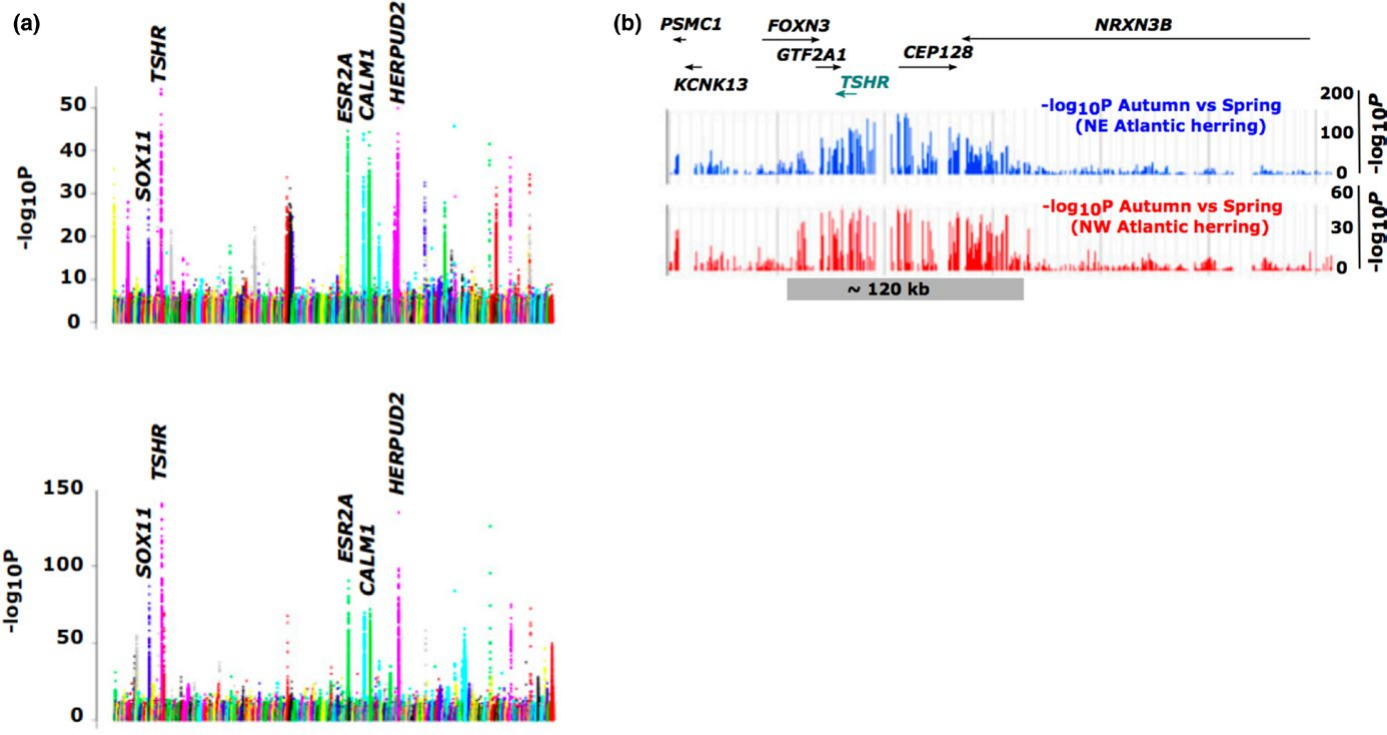
Ecological adaptation in Atlantic herring. (a) Genetic differentiation between autumn and spring‐spawning herring in the northwest Atlantic (upper panel) and in the northeast Atlantic (lower panel). Five genomic regions showing strong differentiation on both sides of the Atlantic Ocean are marked. (b) Genetic differentiation around the *TSHR* locus showing a large haplotype block (~120 kb) shared among northeast and northwest Atlantic populations

Common variation in reproduction patterns in the marine environment include frequency (semelparous or iteroparous), type (batch or total), and season of spawning (Melvin, Stephenson, & Power, 2009). Although these reproductive patterns mostly tend to be fixed within a species, some species show intraspecific variability between populations as part of their local adaptation (Villegas‐Ríos, Alonso‐Fernández, Domínguez‐Petit, & Saborido‐Rey, 2013). One such example is Atlantic herring where different populations spawn in three different seasons—spring, summer and autumn (Martinez Barrio et al., 2016). In a first paper, we compared whole‐genome sequences from three autumn‐spawning populations and 14 spring‐spawning populations from the East Atlantic Ocean and the Baltic Sea and analyzed allele frequencies of each SNP across the genomes and identified ~100 loci that showed significant allele frequency differences between autumn‐ and spring‐spawning populations (Martinez Barrio et al., 2016; Figure 3a, upper panel). The strongest signal overlapped the thyroid stimulating hormone receptor gene (*TSHR),* which has an established role in photoperiodic regulation of reproduction in birds and mammals (Hanon et al., 2015; Nakao et al., 2008; Ono et al., 2008). In the second paper, we further sequenced the genomes of three spring‐spawning and three autumn‐spawning populations from West Atlantic Ocean along the Canadian coast (Lamichhaney et al., 2017). Interestingly, the loci showing the most consistent genetic differentiation between autumn and spring spawners in the northeast and northwest Atlantic populations showed a considerable overlap, with each of the top five genetic signals being replicated (Figure 3a, lower panel). These results indicate that genetic factors affecting timing of reproduction are to a large extent shared among distantly located herring populations. Each of the four major loci that showed genetic differentiation between autumn‐ and spring‐spawning populations was composed of large haplotype blocks with genomic sizes in the range 10–120 kb. The haplotype block including *TSHR* was 120 kb in size, and the two major haplotypes had an estimated coalescence time of about 500,000 years based on their nucleotide divergence (Lamichhaney et al., 2017).

Using similar methods as for screening the structural variants in the ruff genome, we screened for the presence of inversions and other structural variants in autumn‐ and spring‐spawning populations in Atlantic herring. But, we could not detect evidence for any inversion associated with genetic differentiation between populations. In our previous study, we had also identified large divergent haplotype blocks associated with adaptation to the brackish Baltic Sea (Martinez Barrio et al., 2016), but we came to the same conclusion regarding the lack of evidence for the presence of inversions as the case with the loci associated with spawning as discussed here. Therefore, we have concluded that inversions are not a *major* reason for the presence of large haplotype blocks associated with ecological adaptation in Atlantic herring.

## 5 DISCUSSION

The 4.5 Mb inversion in ruff associated with alternate male mating strategies is one of the several examples of “supergenes” maintained by inversions and associated with phenotypic polymorphisms that has been reported in recent years. Other examples include a 400‐kb divergent chromosomal block controlling mimicry in *Heliconius *butterfly (Joron et al., 2011), several megabase inversions showing genomic differentiation between migratory and nonmigratory ecotypes in Atlantic Cod (Berg et al., 2016; Kirubakaran et al., 2016; Sodeland et al., 2016) and a large inversion polymorphism (>100 MB) in white‐throated sparrow linked to variation in plumage, social behavior, and mate choice (Huynh, Maney, & Thomas, 2010).

However, the presence of divergent haplotype blocks does not necessarily imply the presence of an inversion, as was indicated in Atlantic herring populations, where we did not detect inversions linked to divergent haplotype blocks associated with ecological adaptation (Lamichhaney et al., 2017; Martinez Barrio et al., 2016), even though we had access to very similar data as was successfully used to detect the ruff inversion and we examined hundreds of loci. Divergent haplotype blocks could also be the result of genetic hitchhiking during a recent selective sweep (Smith & Haigh, 1974) or natural selection favoring certain haplotype combinations. Haplotype blocks caused by recent selective sweeps are expected to show low sequence divergence and to break down by recombination over time. In contrast, haplotype blocks maintained by some form of balancing selection may evolve over long evolutionary periods and lead to selection for suppressed recombination in the interval even in the absence of an inversion. It is possible that many of the large haplotype blocks (10–200 kb in size) that we identified in the Atlantic herring are of this latter type and have evolved as part of the ecological diversification in this species. This may also be the case for two major loci underlying variation in beak morphology in Darwin's finches involving large haplotype blocks: a 240‐kb region around *ALX1* associated with beak shape variation (Lamichhaney et al., 2015) and a 525‐kb region around *HMGA2* associated with beak size variation (Lamichhaney, Han et al., 2016). In both these cases, sequence divergence between haplotypes was relatively high (estimated coalescence time of about 900,000 years) but there was no evidence for the presence of inversions.

One important difference between the divergent haplotype blocks identified in ruff and Atlantic herring (or Darwin's finches) was their size. The haplotype blocks in the Atlantic herring (10–200 kb) and Darwin's finches (200–500 kb) were much smaller than the one in ruff (4.5 Mb). One might advance a hypothesis about how there might be a scale above which structural rearrangement would be required to suppress recombination. On the other hand, this will likely be dependent on intrinsic recombination rate, which might be variable across systems rather than an absolute physical size. In addition, the methods we used for detecting chromosomal rearrangements were based on short paired‐end read mappings on fragmented genome assemblies. Even though such methods have spurred the unprecedented discovery of structural variants in a range of species (Wellenreuther & Bernatchez, 2018), the scale of false‐positives and false‐negatives produced by these methods is still high and these methods are blind to the identification of inversions if the breakpoints are located in repetitive regions (Lucas Lledó & Cáceres, 2013). Hence, we cannot exclude the possibility that some of the divergent haplotype blocks detected in Atlantic herring or Darwin's finches are in fact caused by inversions, but we did not have enough power to detect them due to the abovementioned limitations.

Current advancements in “third generation” sequencing technologies include longer sequencing reads such as PacBio (Rhoads & Au, 2015), Oxford Nanopore (Jain, Olsen, Paten, & Akeson, 2016), or chromosomal level genome assemblies based on mapping long‐range interactions (Hi‐C) (Belton et al., 2012). The power to detect inversions (and other chromosomal rearrangements) in genomes has been dramatically improved with the arrival of these single‐molecule long‐read sequencing methods (Sedlazeck et al., 2018) and provide exciting opportunities to correctly identify inversions and other chromosomal rearrangements (Merker et al., 2017). In fact, we are currently sequencing the genomes of herring, ruff and Darwin's finches using these “third generation” long‐read sequencing methods and therefore expect to have more conclusive evidence concerning the presence/absence of inversions associated with adaptive traits in these systems in the near future.

This comparative review based on our previously published datasets of three species system indicate that divergent haplotype blocks associated with phenotypic variation do not necessarily imply the presence of an inversion. However, we also note that this paper only evaluates large haplotype blocks and the presence of inversion by comparing three previously published well‐characterized datasets of genomic regions associated with phenotypic changes. Additional datasets from other species system generated using similar methods described in this paper will be required to come into a general conclusion on how important inversions are for explaining haplotype blocks with strong linkage disequilibrium in natural populations.

## CONFLICT OF INTEREST

None declared.

## AUTHOR CONTRIBUTION

Both authors (SL & LA) planned the organization and themes of the paper. SL led the writing of the paper, with substantial writing from LA. Both authors edited and approved the final text.

## Data Availability

This article was utilized previously published data for this comparative study. No additional data were generated.
